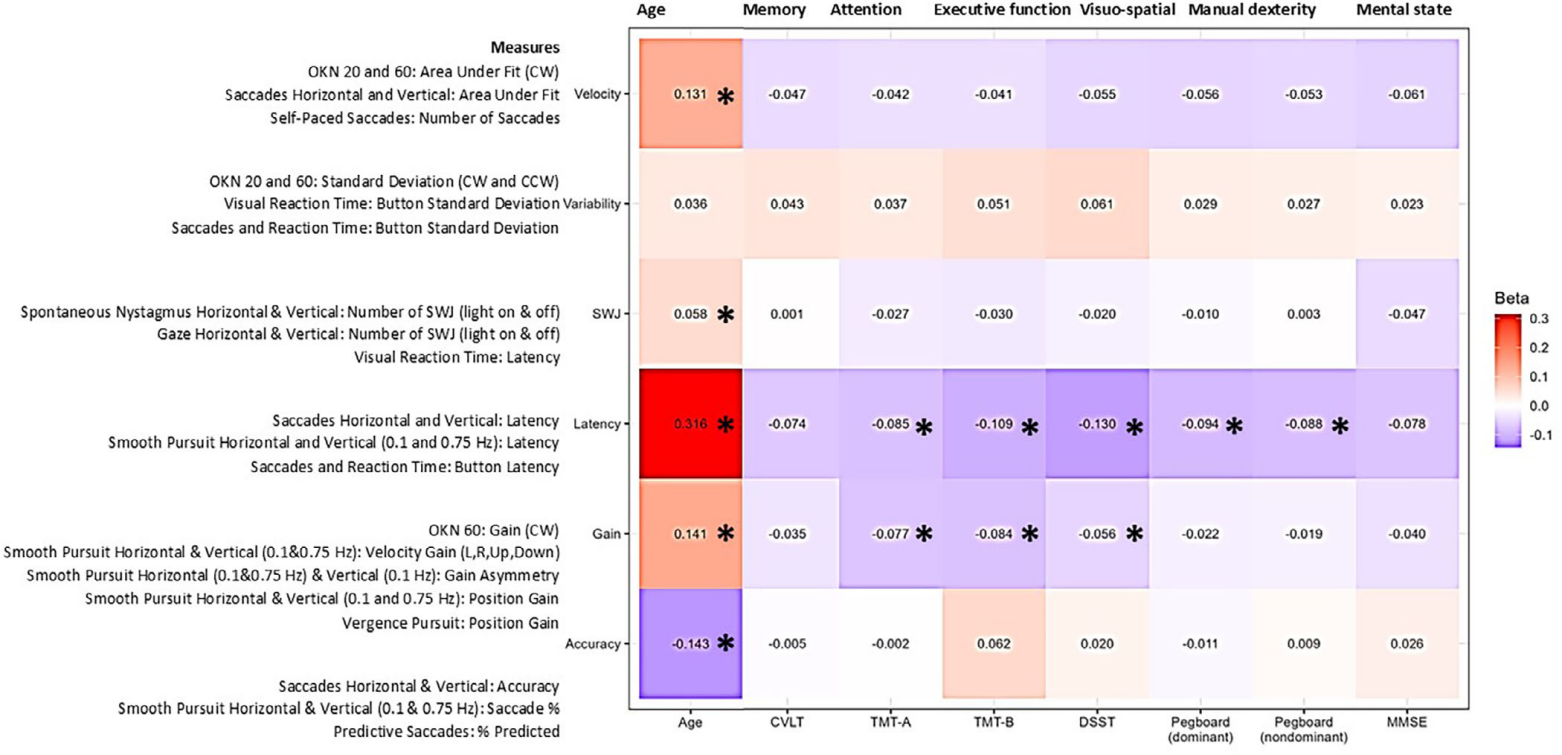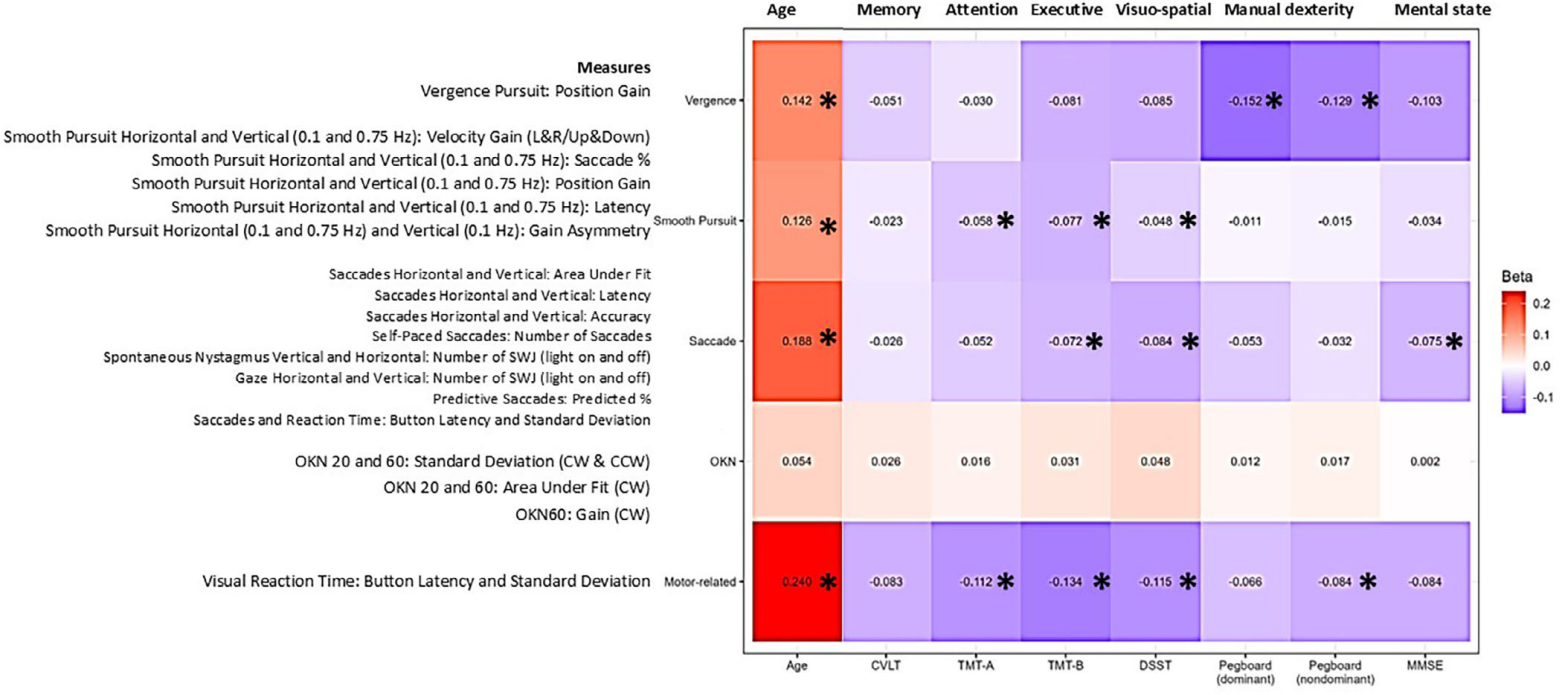# Eye Movements Metrics Links to Cognitive Impairment and Cognition

**DOI:** 10.1002/alz70856_100612

**Published:** 2025-12-24

**Authors:** Qu Tian, Carla Hamwi, Yang An, Susan M. Resnick, Luigi Ferrucci

**Affiliations:** ^1^ National Institute on Aging, Baltimore, MD, USA; ^2^ National Institute on Aging Intramural Research Program, Baltimore, MD, USA; ^3^ Brain Aging and Behavior Section, National Institute on Aging, NIH, Baltimore, MD, USA; ^4^ National Institute on Aging, National Institutes of Health, Baltimore, MD, USA; ^5^ Translational Gerontology Branch, National Institute on Aging, NIH, Baltimore, MD, USA

## Abstract

**Background:**

Ocular motor function, or eye movement, serves as a vital indicator of brain health and behavior. Recent advancements in technology have made eye tracking a powerful, quantitative, non‐invasive, and portable tool for studying cognition, brain health, and related diseases. This method allows for objective measurement without verbal or motor responses from the participant. However, it remains unclear which eye movement metrics are most sensitive or specific to various cognitive domains. Existing studies have focused on patients with neurodegenerative diseases or limited to selected eye movement metrics and cognitive assessments. In this study, we aimed to explore the relationships between a diverse array of eye movement metrics with cognitive impairment and neuropsychological performance, in well‐characterized community‐dwelling adults.

**Methods:**

In 544 Baltimore Longitudinal Study of Aging participants (mean age=71 years[28‐99], 58%women, 25%Black, 5%cognitive impairment/dementia), we analyzed 51 eye movement measures (<5% missing) via a portable eye‐tracking device (Neurolign Dx100). We extracted principal components (PCA) on eye movement categories (saccade, smooth pursuit, motor‐related, optokinetic nystagmus, and vergence) and measurement types (accuracy, gain, latency, velocity, square‐wave jerks, and variability). Cognitive impairment was determined by consensus case conferences, using clinical and neuropsychological evaluations. We examined associations with cognitive impairment and various cognitive functions using logistic and linear regression, respectively, adjusted for age and sex.

**Results:**

Saccadic eye movements and speed‐related measures (latency, velocity, variability) were associated with cognitive impairment (odds ratio=1.308, 1.427, 1.987, 0.727, respectively, all *p* <0.05). Saccades were also associated with the Mini‐Mental State Exam, executive function (Trail Making Test (TMT)‐part B), and visuo‐perceptual speed (Digit Symbol Substitution Test). Smooth pursuit, visual reaction time, and measures of gain and latency were associated with attention (TMT‐A), executive function, and visuo‐perceptual speed. Vergence, visual reaction time, and latency were related to manual dexterity (Pegboard performance)(all Bonferroni‐adjusted *p* <0.05). Other categories or types of measures were not associated with cognitive impairment or cognition.

**Conclusion:**

Saccades and speed‐related eye movement metrics link to cognitive impairment. Specific eye movement categories and measures are associated with selected cognitive domains. Longitudinal studies are needed to determine whether these eye movement metrics can serve as early predictors of future cognitive decline and brain pathology.